# Supplementary material for: Antimicrobial Spectrum of Titroleane™: A New Potent Anti-Infective Agent
Source: Antibiotics (Basel). 2020 Jul 8;9(7):391. doi: 10.3390/antibiotics9070391 (PMC7400619; doi:10.3390/antibiotics9070391)
Supplement: Supplementary file 1 [file antibiotics-09-00391-s001.pdf]

Supplementary materials

Figure S1

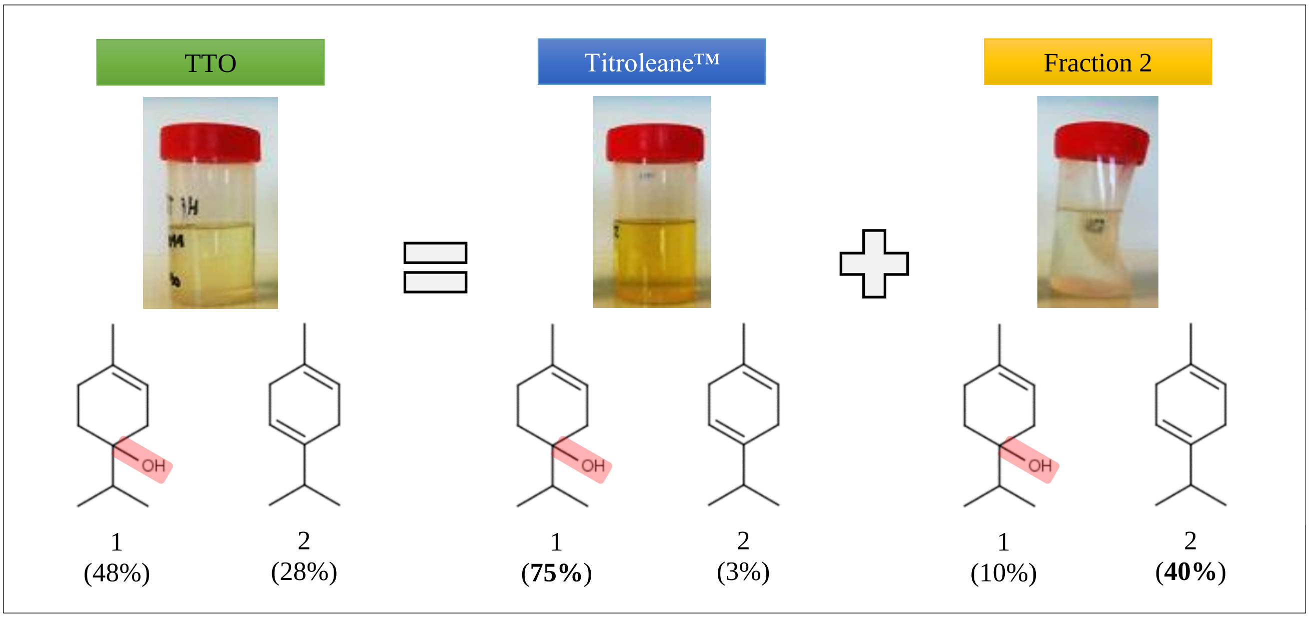

Figure S1. Extracts after two-month storage. Presentation of plastic containers with TTO, Titroleane™ or Fraction 2 after two-month storage. The two main molecules of TTO are also represented with their concentrations in percent in each extract. 1: terpinen-4-ol, 2: γ-terpinene.
